# The Role of Sch9 and the V-ATPase in the Adaptation Response to Acetic Acid and the Consequences for Growth and Chronological Lifespan

**DOI:** 10.3390/microorganisms9091871

**Published:** 2021-09-03

**Authors:** Marie-Anne Deprez, Jeroen M. Maertens, Lisbeth Olsson, Maurizio Bettiga, Joris Winderickx

**Affiliations:** 1 Functional Biology, KU Leuven, Kasteelpark Arenberg 31, 3000 Leuven, Belgium; marieanne.deprez@kuleuven.be; 2 Department of Biology and Biological Engineering, Division of Industrial Biotechnology, Chalmers University of Technology, SE-412 96 Gothenburg, Sweden; jeroen.maertens@chalmers.se (J.M.M.); lisbeth.olsson@chalmers.se (L.O.); maurizio.bettiga@chalmers.se (M.B.)

**Keywords:** acetic acid stress, nutrient signalling, pH homeostasis, growth, chronological lifespan, lipidomics

## Abstract

Studies with *Saccharomyces cerevisiae* indicated that non-physiologically high levels of acetic acid promote cellular acidification, chronological aging, and programmed cell death. In the current study, we compared the cellular lipid composition, acetic acid uptake, intracellular pH, growth, and chronological lifespan of wild-type cells and mutants lacking the protein kinase Sch9 and/or a functional V-ATPase when grown in medium supplemented with different acetic acid concentrations. Our data show that strains lacking the V-ATPase are especially more susceptible to growth arrest in the presence of high acetic acid concentrations, which is due to a slower adaptation to the acid stress. These V-ATPase mutants also displayed changes in lipid homeostasis, including alterations in their membrane lipid composition that influences the acetic acid diffusion rate and changes in sphingolipid metabolism and the sphingolipid rheostat, which is known to regulate stress tolerance and longevity of yeast cells. However, we provide evidence that the supplementation of 20 mM acetic acid has a cytoprotective and presumable hormesis effect that extends the longevity of all strains tested, including the V-ATPase compromised mutants. We also demonstrate that the long-lived *sch9*Δ strain itself secretes significant amounts of acetic acid during stationary phase, which in addition to its enhanced accumulation of storage lipids may underlie its increased lifespan.

## 1. Introduction

Weak organic acids are commonly used as preservatives due to their growth inhibiting effect, but these weak acids are also produced in small amounts by yeast cells as a by-product during fermentative growth [1]. However, when yeast cells reach the diauxic shift and switch to a respiratory metabolism, acetic acid is taken up from the medium and used as a carbon source. This uptake is facilitated by the de-repression and induction of several plasma membrane acetate permeases [2,3]. Once inside the cell, acetate is converted to acetyl-CoA and then further metabolized [4]. In addition to uptake via the acetate transport systems, the undissociated form of acetic acid is able to passively diffuse through the membranes [2]. After cellular entry, most of the acetic acid dissociates at the neutral intracellular pH (pKa 4.8), causing intracellular acidification and inhibition of fermentative enzymes, which eventually can lead to growth inhibition and even the induction of programmed cell death [5,6,7,8,9,10]. Thus, it is not the total external concentration but rather the concentration of the undissociated form of acetic acid that appears to be the main determinant for its detrimental effects on cell growth [2,11].

Yeast cells activate several adaptive responses to facilitate a certain tolerance to acetic acid [1,12,13]. Firstly, since acetic acid causes intracellular acidification, the plasma membrane proton pump Pma1 and the vacuolar V-ATPase play a crucial role in counteracting the changes in intracellular pH and membrane proton gradients [14,15,16]. Indeed, mutant strains that fail to maintain pH homeostasis, such as the *VMA* mutants that lack functional V-ATPases, or the *pma1-007* mutant that has reduced Pma1 activity, make cells more susceptible to sublethal concentrations of acetic acid [14,17,18]. Conditions known to reduce the activity of these ATP-dependent proton pumps, such as a short glucose starvation, also lead to more severe changes in intracellular pH when cells are subjected to acetic acid treatment [14,19]. Secondly, intracellular acetate triggers the induction of several transcriptional networks controlled by transcription factors such as Haa1, Msn2/4, and Gis1 [17,18,20,21,22], leading to the enhanced expression of stress responsive genes and different multidrug resistance transporters (Tpo2, Tpo3, Azr1, Aqr1, and Pdr18) that have all been linked to the export of the acetate counter ions (CH_3_COO^−^) [20,23,24]. In addition, cells display a metabolic reprogramming controlled by the nitrogen signalling kinases Tor1 and Gcn2 in order to adapt to the limiting nutrient and amino acid supply caused by acetic acid stress [25,26]. Finally, more recent studies implicated changes in lipid biosynthesis and the rearrangements in cellular lipid composition as another important aspect by which yeast cell adapt to weak acid stress. These rearrangements include changes in abundance of glycerophospholipids (GPL) and sphingolipid classes, adaptations in fatty acid chain length and saturation, and changes in sterol abundance [27,28,29,30].

High concentrations of acetic acid, i.e., 80–120 mM at pH 3, lead to an apoptotic-like regulated cell death, while even higher concentrations were shown to trigger a necrotic type of cell death. The apoptotic-like cell death process is accompanied by the translocation of cytochrome c from mitochondria and accumulation of reactive oxygen species (ROS) [31]. Therefore, it is not surprising that activation of the retrograde signalling pathway, a process involving the mitochondrial dysfunction sensor Rtg2 and the protein kinase Hog1, and the activation of catalase and superoxide dismutase gives rise to acetic acid resistance [13,32,33,34,35,36,37]. Interestingly, the loss of the nutrient signalling kinases Sch9 and TORC1 also result in acetic acid resistance [11,25]. For Sch9, this effect is dependent on the STRE and PDS transcription factors Msn2/4 and Gis1 [11], which are also responsible for the expression of superoxide dismutases and catalases. In agreement with this finding, addition of higher-than-physiological concentrations of acetic acid to the medium, i.e., 100 mM at pH 3.7, completely represses activation of STRE and PDS regulons [38]. Besides acting via the Sch9 effector branch, TORC1 was also shown to affect acetic acid-induced apoptotic-like signalling via the Tap42-Pph21/22 effector branch, thereby acting in conjunction with the Gcn2/Gcn4 general amino acid control system to counteract the acetic acid-induced amino acid starvation and the reduced translation efficiency [25].

Sublethal concentrations of acetic acid have been shown to induce chronological aging [11,38,39]. In fact, it has been stated that acetic acid could the primary mechanism of chronological aging in yeast under normal growth based on the observation that conditions that extend lifespan, such as calorie restriction, growth of cells on non-fermentable carbon sources, or transferring cells to water limit the accumulation of extracellular acetic acid [11]. The latter was suggested to mimic the buffering of the medium [11], a condition that is also known to enhance longevity [40,41]. However, this view has been challenged in a study showing that strains carrying mutations in genes involved in acetic acid metabolism do not affect medium acidification or chronological aging [38]. This study also demonstrated that the enhanced longevity phenotype of *tor1*Δ and *sch9*Δ strains is linked to their ability to activate an alternative metabolic pathway upon the depletion of glucose that allows to faster consume ethanol, which has pro-aging characteristics, and to utilize acetic acid for the production and storage of the protective reserve carbohydrate trehalose [38]. Similar observations were also made for the long-lived *sir2*Δ strain [42].

Previously, we reported data that linked the enhanced chronological lifespan of the *sch9*Δ strain to changes in sphingolipid and ceramide biosynthesis as well as to alterations in pH homeostasis and especially the presence of more acidic vacuoles [41,43]. Given the importance of cellular lipid composition and pH homeostasis in the cellular response to acetic acid stress, as mentioned above, we decided to perform a systematic analysis and to compare different aspects of acetic acid tolerance and toxicity between the BY4741 wild-type strain and the isogenic *sch9*Δ, *vma2*Δ and *sch9*Δ*vma2*Δ mutants. The latter two strains are lacking a functional V-ATPase and they were included due to the complex interplay between nutrient signalling, lipid biosynthesis and V-ATPase activity [41,44,45,46,47].

## 2. Materials and Methods

### 2.1. Yeast Strains and Growth Conditions

The *Saccharomyces cerevisiae* wild type (WT) BY4741 *(MATa his3*Δ*1 leu2*Δ*0 met15*Δ*0 ura3*Δ*0*) and the isogenic *sch9::NatMX*, *vma2::KanMX* and *sch9::NatMX vma2::KanMX* mutants have been described previously [41]. Cells were grown at 30 °C in standard rich medium containing 20 g/L peptone and 10 g/L yeast extract (YP) or in synthetic medium (S) containing 0.5% (NH_4_)_2_SO_4_, 1.9 g/L yeast nitrogen base without amino acids, supplemented with either synthetic drop-out mixtures or synthetic complete mixture as required (Formedium), and 20 g/L glucose (D).

For the long-term acetic acid treatment experiments, cells were pre-grown in synthetic complete medium (SDc) buffered at pH 5.0 with 50 mM MES. After 24 h of growth, cultures were diluted to OD_600nm_ 0.1 in SDc with 50 mM potassium hydrogen phthalate pH 5, supplemented with 0, 2, 20 or 150 mM acetic acid. Starting from this timepoint (TP0), culture samples were taken at predetermined time points (6 h, 24 h, 48 h, 6 days, 12 days, 16 days, 20 days) for subsequent analysis by different methods.

### 2.2. Lipidomics

Yeast cultures were grown to OD 1 and of these cultures 20 OD units of cells were pelleted and snap frozen in liquid nitrogen. The cells were broken by vigorous vortexing with glass beads, followed by clearance of the sample by centrifugation. At that time a sample was taken to determine the protein concentration using a NanoDrop spectrophotometer (Thermo Scientific, Uppsala, Sweden), which was used to calibrate the cell extracts. The snap frozen cell extracts were then transported on dry ice to Lipotype GmbH (Tatzberg, Germany) where shotgun lipidomics analyses were performed on total lipids. For each strain, three independent samples were analyzed.

The protocols for mass spectrometry-based lipid analysis performed by Lipotype GmbH have been described previously [48,49]. Lipids were extracted using a two-step chloroform/methanol procedure [49]. Briefly, samples were spiked with an internal lipid standard mixture containing: cytidine diphosphate diacylglycerol (CDP-DAG) 17:0/18:1, ceramide (Cer) 18:1;2/17:0, diacylglycerol (DAG) 17:0/17:0, lysophosphatidate 17:0 (LPA), lyso-phosphatidylcholine 12:0 (LPC), lysophosphatidylethanolamine 17:1 (LPE), lyso-phosphatidylinositol 17:1 (LPI), lysophosphatidylserine 17:1 (LPS), phosphatidate (PA) 17:0/14:1, phosphatidylcholine (PC) 17:0/14:1, phosphatidylethanolamine (PE) 17:0/14:1, phosphatidylglycerol (PG) 17:0/14:1, phosphatidylinositol (PI) 17:0/14:1, phosphatidylserine (PS) 17:0/14:1, ergosterol ester (EE) 13:0, triacylglycerol (TAG) 17:0/17:0/17:0, stigmastatrienol, inositolphosphorylceramide (IPC) 44:0;2, mannosyl-inositolphosphorylceramide (MIPC) 44:0;2 and mannosyl-di(inositolphosphoryl)-ceramide (M(IP)_2_C) 44:0;2. After extraction, the organic phase was transferred to an infusion plate and dried in a speed vacuum concentrator. First-step dry extract was re-suspended in 7.5 mM ammonium acetate in chloroform/methanol/propanol (1:2:4, V:V:V) and second-step dry extract in a 33% ethanol solution of methylamine in chloroform/methanol (0.003:5:1; V:V:V). All liquid handling steps were performed using a Hamilton Robotics STARlet robotic platform (Hamilton, Gräfelfing, Germany) with the Anti Droplet Control feature for organic solvent pipetting.

Samples were analyzed by direct infusion on a QExactive mass spectrometer (MS) (Thermo Scientific, Munich, Germany) equipped with a TriVersa NanoMate ion source (Advion Biosciences, Ithaca, NY, USA). Samples were analyzed in both positive and negative ion modes with a resolution of R_*m*/*z*=200_ = 280,000 for MS and R_*m*/*z*=200_ = 17,500 for MS/MS experiments, in a single acquisition. MS/MS was triggered by an inclusion list encompassing corresponding MS mass ranges scanned in 1 Da increments [50]. Both MS and MS/MS data were combined to monitor EE, DAG. and TAG ions as ammonium adducts, PC as an acetate adduct, and CL, PA, PE, PG, PI. and PS as deprotonated anions. MS only was used to monitor LPA, LPE, LPI, LPS, IPC, MIPC, and M(IP)_2_C as deprotonated anions, and Cer and LPC as acetate adducts.

Data were analyzed with in-house developed lipid identification software based on LipidXplorer ([51], Lypotype GmbH (Tatzberg, Germany)). Data post-processing and normalization were performed using an in-house developed data management system. The identified lipid molecules were quantified by normalization to a lipid class-specific internal standard. Only lipid identifications with a signal-to-noise ratio >5, and a signal intensity 5-fold higher than in corresponding blank samples, were considered for further data analysis. All analysis was performed using lipid data normalized to the total lipid content yielding mol% per sample.

### 2.3. Chronological Lifespan Analysis

Cell death and the accumulation of reactive oxygen species (ROS) was measured at regular time points with flow cytometry (Guava easyCyte 8HT, Merck Millipore, Overijse, Belgium). In order to do so, cells were co-stained with 30 nM SYTOX™ green (Invitrogen, Merelbeke, Belgium) and 5 µg/mL dihydroethidium (DHE). 5000 cells per biological replicate per condition were measured. Analysis of the different cell populations (SYTOX−/DHE−, SYTOX−/DHE+, SYTOX+) was done using GuavaSoft v3.3 (Merck Millipore, Overijse, Belgium). At least three independent samples were analysed for each strain.

### 2.4. Medium Analysis

Medium was sampled at regular time points to measure pH and determine the concentration of certain metabolites (such as glucose, ethanol and acetic acid) throughout growth and lifespan. The samples were filtered (0.2 µm PFTE filter (Sigma-Aldrich, Stockholm, Sweden)) and analysed using a Jasco LC-4000 high-performance liquid chromatography system (JASCO international Co. ltd, Tokyo, Japan). The system is equipped with an AS-4150 autosampler, a PU-4180 pump, a CO-4061 column oven, an UV-4075 detector and a refractive index (RI-4030) detector. The liquid phase consisted of an isocratic concentration of sulfuric acid (5 mM) in water and had a constant flow rate of 0.8 mL/min. The column used was an analytical Rezex ROA Organic acid H^+^ column (Phenomenex, Aschaffenburg, Germany) and was maintained at 80 °C. Analysis was performed using Chromnav software (JASCO international CO. ltd, Tokyo, Japan) and concentrations were determined using standard calibrations.

### 2.5. Intracellular pH Measurements

The overall protocol for pH measurements has been described previously [41]. For each measurement, at least three independent samples were analysed. For the long-term steady-state cytosolic pH measurements, cells transformed with the pYES2-pACT1-pHluorin plasmid (kindly provided by G. Smits) were pre-grown on SD-URA buffered at pH 5 with 50 mM MES. After 48 h, all cultures were equalized to a start OD_600nm_ of 0.5 in low fluorescence (loflo) medium, buffered with 50 mM potassium hydrogen phthalate pH 5, supplemented with 0, 2, 20, or 150 mM acetic acid. Cells were sampled immediately after 6 h, 24 h and 48 h of growth, and concentrated in the growth medium. For the short-term cytosolic and vacuolar pH measurements with an acetic acid pulse, cells were transformed with the pYES2-pACT1-pHluorin plasmid or with the corresponding empty vector control (pRS416) respectively. The cells were grown to mid-log phase on SD-URA buffered at pH 5 with 50 mM phthalate. After concentrating the cells in loflo medium with 50 mM phthalate pH 5, they were loaded with 50 µM BCECF-AM (Merck Millipore, Overijse, Belgium) for vacuolar pH measurements [41]. Cells were washed twice in 50 mM phtalate pH 5 in case measurements were done in buffer or with loflo medium with 50 mM phthalate pH 5, in case measurements were done in medium.

Fluorescence was measured over time at 510 nm after excitation at 440 nm and 485 nm for BCECF-AM measurements and 390 nm and 485 nm for pHluorin measurements with a Fluoroskan Ascent FL Microplate Fluorometer (Thermo Scientific, Merelbeke, Belgium). For each measurement a calibration curve was constructed using a calibration buffer as previously described [52], but lacking monensin and nigericin, as these compounds were found to be dispensable. For the long-term pH data, the represented data are the average pH from 1 to 2 h after the start of the measurement.

### 2.6. Analysis of Acetic Acid Diffusion

Acetic acid uptake measurements were performed as described before [53]. Triplicate yeast cultures were grown overnight to mid-log phase. The cells were washed twice in ice-cold 50 mM potassium hydrogen phthalate buffer pH 5 and stored on ice until analysis (but no longer than 3 h).

A predetermined amount of [1-^14^C] acetic acid (20 nCi/µL final concentration, 0.36 mM acetic acid) was mixed with an amount of non-radiolabelled acetic acid (0.2, 2, 20, and 150 mM acetic acid). This resulted in specific activities of 72,300, 16,400, 1960 and 270 DPM/mmol acetic acid. Each assay was performed by addition of 60 µL of cell suspension to 180 µL of 50 mM potassium hydrogen phthalate buffer (pH 5.0) and incubation in a 30 °C water bath for 4 min. Addition of 60 µL of the acetic acid mixture was followed by sampling three times as quickly as possible (ca. 10 s, 45 s and 80 s) and subsequent sampling at 5 and 10 min. The mixture was kept at 30 °C as much as possible. Sampling was performed by taking 50 µL from the assay mixture and immediately adding it to 10 mL of ice-cold stop solution at the same (unlabelled) acetic acid concentration as the assay. The mixture was filtered through a Whatman GF/C filter (25 mm diameter, Whatman, Maidstone, UK). The filters were then washed with 10 mL of ice-cold stop solution at the same (unlabelled) acetic acid concentration. The filters were placed in scintillation vials containing 10 mL Emulsifier-Safe™ scintillation liquid (Perkin Elmer, Groningen, The Netherlands) and shaken thoroughly.

The samples were analyzed by measuring the radioactive decay using a Wallac Guardian 1414 liquid scintillation counter (Perkin Elmer, Upplands Väsby, Sweden). The specific activities were determined by adding a small amount of acetic acid mixture directly to a Whatman GF/C filter paper and placing it into scintillation vials containing 10 mL Emulsifier-Safe™ scintillation liquid. Blanks were prepared by adding cell suspension directly to ice cold stop solution and filtering it over a fresh Whatman GF/C filter and inserting the filter into scintillation vials containing 10 mL Emulsifier-Safe™ scintillation liquid. Natural background radiation was measured using only 10 mL Emulsifier-Safe™ scintillation liquid. The intracellular acetic acid concentration was calculated assuming a cell volume of 2 µL/mg dry weight. The measured decay was linear in the concentration range evaluated, and no quenching effects from the sample matrix were observed.

## 3. Results

### 3.1. Loss of Sch9 or the V-ATPase Impacts on Lipid Homeostasis

**Figure 1:**
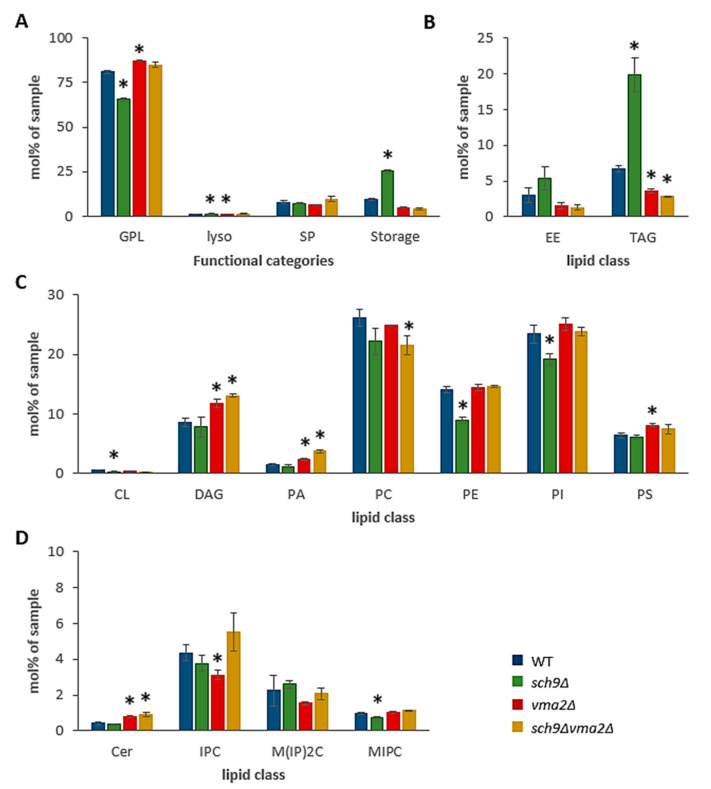
Sch9 and V-ATPase are involved in lipid homeostasis. Shown are the lipid concentrations for the different functional categories (**A**) and the different lipid classes within the functional categories: i.e., storage lipids (**B**), glycerophospholipids (**C**), and sphingolipids (**D**). As indicated, the data are shown for the BY4741 WT strain (blue bars) and the isogenic mutant strains *sch9*Δ (green bars), *vma2*Δ (red bars) and *sch9*Δ*vma2*Δ (ochre bars). Results are given as mean ± SD of 3 biological replicates (*n* = 3), * *p* < 0.05 as tested with *t*-tests in comparison to the WT. Nomenclature: GPL, glycerophospholipids; lyso, lysolipids, SP, sphingolipids; EE, ergosterol esters; TAG, triacylglycerol; CL, cardiolipin; DAG, diacylglycerol; PA, phosphatidic acid; PC, phosphatidylcholine; PE, phosphatidylethanolamine; PI, phosphatidylinositol; PS, phosphatidylserine; Cer, ceramide; IPC, inositolphosphorylceramide; MIPC, mannosyl-inositolphosphorylceramide; M(IP)_2_C, mannosyl-(diphosphoinositol)-ceramide.

To identify effects of lipid metabolism and its implications for acetic acid diffusion over the plasma membranes, we performed a lipidomic shotgun analysis on WT, *sch9*Δ, *vma2*Δ and *sch9*Δ*vma2*Δ cells grown to mid-exponential phase on glucose-containing medium. This analysis revealed some interesting differences between strains as shown when comparing the functional categories of total lipids (Figure 1A). The *sch9*Δ strain has a distinct increase in storage lipids, mostly due to a strong upregulation of triacylglycerol (TAG), which is compensated by a reduction in overall glycerophospholipids (GPL) (Figure 1A,B). Further breakdown of the GPL lipid classes displays a reduction of all classes in the *sch9*Δ strain in comparison to the WT strain, though only the reduction of phosphatidylethanolamine (PE), phosphatidylinositol (PI) and the mitochondrial lipid cardiolipin (CL) was significant (Figure 1C). In contrast, the TAG fraction of the *vma2*Δ and *sch9*Δ*vma2*Δ strain is significantly reduced compared to the WT, which in turn is compensated by an increase in GPL (Figure 1A,B). The *vma2*Δ and *sch9*Δ*vma2*Δ strains displayed an increase in the levels of diacylglycerol (DAG), phosphatidic acid (PA) and phosphatidylserine (PS). In addition, and similar as the *sch9*Δ strain, the *sch9*Δ*vma2*Δ strain was also characterized by a significant decrease in phosphatidylcholine (PC) (Figure 1C).

**Figure 2:**
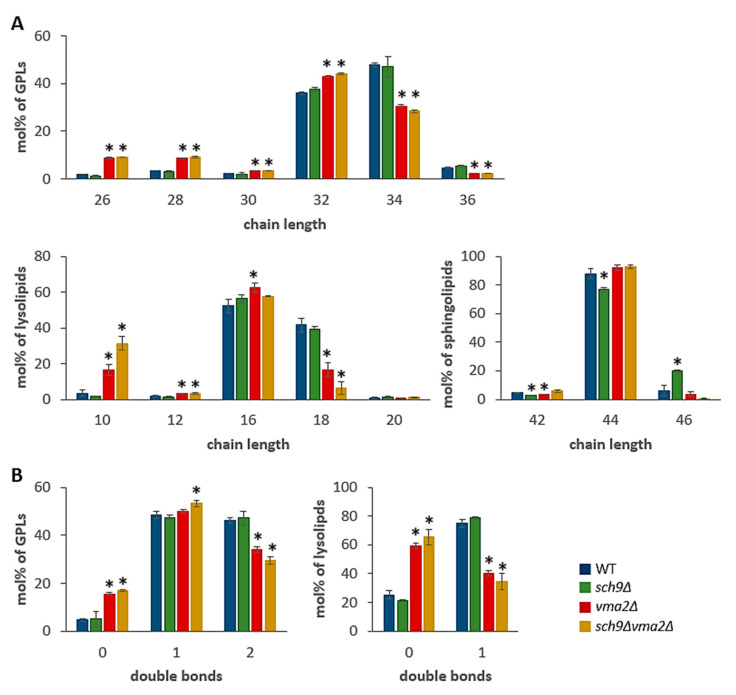
Loss of the V-ATPase leads to shorter chain length and higher saturation. (**A**) Fatty acid chain length in the functional categories of glycerophospholipids (GPLs), lysolipids and sphingolipids. (**B**) Number of double bonds in the fatty acid chains of the functional categories GPLs and lysolipids. The data are given for the BY4741 WT strain (blue bars) and the isogenic mutant strains *sch9*Δ (green bars), *vma2*Δ (red bars) and *sch9*∆*vma2*Δ (ochre bars) as indicated. Results are shown as mean ± SD of 3 biological replicates (*n* = 3), * *p* < 0.05 as tested with *t*-tests in comparison to the WT.

A more detailed analysis of the sphingolipid classes revealed a slight decrease of the complex sphingolipid MIPC in the *sch9*Δ strain, which might be due to a lower IPC content, as reported before in a study describing the role of Sch9 in the sphingolipid rheostat, an important determinant for stress tolerance and lifespan of yeast cells [43]. Albeit in the current lipidomics analysis the IPC level were indeed reduced in the *sch9*Δ mutant, statistical analysis showed this not to be significantly different when compared to the WT strain. The *vma2*Δ and *sch9*Δ*vma2*Δ strains, on the other hand, displayed significantly higher ceramide levels than the WT strain (Figure 1D), indicative that loss of the V-ATPase also impacts on the sphingolipid rheostat and the dynamic balance between the various sphingolipid metabolites. Although higher complex sphingolipid levels have been linked to acetic acid tolerance in *Z. bailli* [30,54], their physiological significance in cell wall remodeling and acetic acid tolerance in *S. cerevisiae* remains unclear. Nonetheless, the membrane permeability for acetic acid is known to be dependent on membrane thickness and lipid chain-packing properties for which fatty acid chain length and saturation are important determinants [55]. The fatty acid chain length and saturation of the *sch9*Δ strain are overall very similar to that of the WT, with one notable exception: sphingolipids (SP) have elongated lipid chains in the *sch9*Δ strain. For the *vma2*Δ and *sch9*Δ*vma2*Δ strain, a clear trend towards shorter fatty acid chain length and higher saturation is notable in GPL and lysolipids (Figure 2A,B). The latter observation could indicate a higher acetic acid membrane permeability of these strains.

### 3.2. Loss of Sch9 or the V-ATPase Leads to Reduced Intracellular Acetic Acid Accumulation

**Figure 3:**
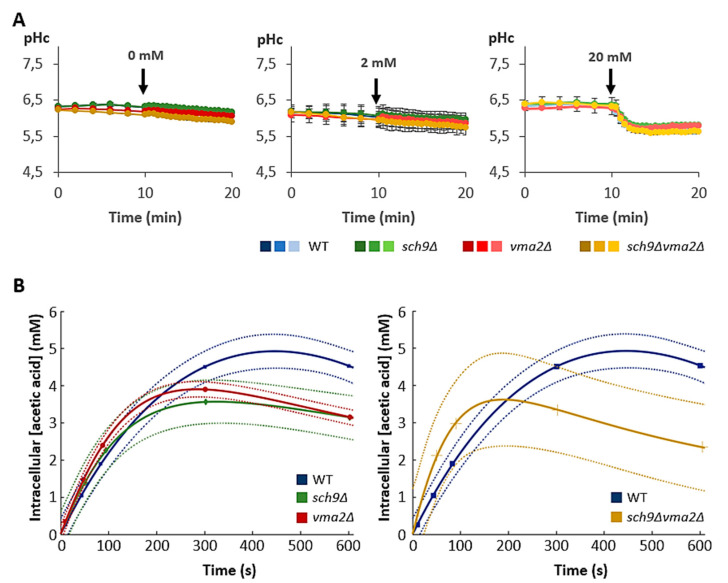
Passive diffusion of acetic acid into yeast cells. Cells expressing cytosolic pHluorin were grown to mid-log phase in medium without acetic acid. (**A**) After a short starvation on buffer, an equal volume of concentrated acetic acid was added to each culture to reach a final concentration of 0, 2 or 20 mM. The acetic acid pulse is indicated by the black arrow. Results are shown as mean ± SD of 3 biological replicates (*n* = 3). (**B**) After a short starvation in buffer, 0.2 mM C14-labelled acetic acid was pulsed to the cells (0 min) and the uptake was monitored during 10 min. Results are shown with 95% confidence intervals in dotted lines. The data are given for the BY4741 WT strain (blue lines, curve fit R^2^: 0.99) and the isogenic mutant strains *sch9*Δ (green lines, R^2^: 0.97), *vma2*Δ (red lines, R^2^: 1.0) and *sch9*Δ*vma2*Δ (ochre lines, R^2^: 0.88) as indicated.

We used two separate approaches to elucidate if acetic acid influx via passive diffusion is different between the strains. First, we measured the cytosolic pH in cells expressing a pH-sensitive pHluorin marker (Figure 3A). These measurements were performed after a short starvation in phthalate buffer pH 5 lacking all nutrients before the acetic acid pulse was given. The ATP-dependent proton pumps, i.e., Pma1 and the V-ATPases, are mostly inactive in these conditions, and therefore cells can no longer mask the intracellular pH changes due to acetic acid influx. As a consequence, the observed cytosolic acidification can be used as a proxy for the amount of acetic acid diffusion over the cell membrane.

Although addition of 0 mM or 2 mM acetic acid led to no detectable intracellular acidification, a clear cytosolic pH drop could be noticed after addition of 20 mM acetic acid in all strains. The extent of intracellular acidification was, however, the same in all tested strains, which suggests that there is no difference in intracellular acetic acid accumulation between these strains (Figure 3A). However, this technique might not be sensitive enough to detect small differences in cellular acetic acid entry at the concentrations tested. Therefore, we also monitored acetic acid uptake dynamics based on intracellular C^14^-labelled acetic acid quantifications. These measurements were again performed in phthalate buffer pH 5. To identify differences in dynamics, we first added a very low concentration of 0.2 mM acetic acid to the cells. As shown, these measurements indicated that when reaching steady-state levels at approximately 5 min after acetic acid addition, the *sch9*Δ*, vma2*Δ and *sch9*Δ*vma2*Δ cells all accumulated less acetic acid as compared to WT cells (Figure 3B). Upon supplementation of higher amounts of acetic acid (2 mM and 20 mM), this difference in the acetic acid accumulation was sustained in all mutant strains, indicating that both Sch9 and the V-ATPase are important to maintain the acetic acid influx (supplemental Figure S1). When the acetic acid uptake rates were plotted (supplemental Figure S2), we noted that the mutant strains displayed a similar tendency of a faster initial uptake that quickly declined to levels below that observed in the WT cells when approaching the steady state. Especially in the *sch9*Δ*vma2*Δ strain the initial acetic acid uptake was markedly higher than that of the WT (Figure 3B; supplemental Figure S2), again suggesting a role of Sch9 and the V-ATPase in determining the membrane permeability. Whether the role of the V-ATPase relates to the observation that this proton pump is involved in the regulation of membrane thickness and rigidity remains to be further investigated. Furthermore, it should be noted that the differences in initial uptake dynamics between the strains became less pronounced when higher acetic acid concentrations were given to the cells (supplemental Figure S1).

### 3.3. A Functional V-ATPase Is Required to Adapt to Conditions of Extreme Acid Stress

**Figure 4:**
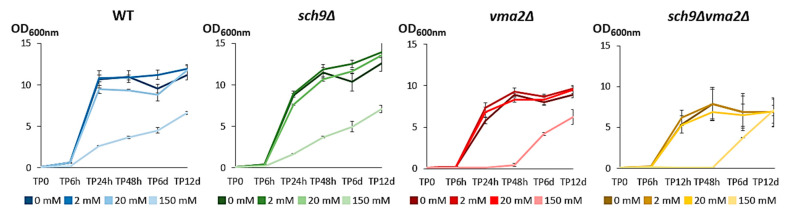
The effect of acetic acid on growth of the BY4741 WT, *sch9*Δ, *vma2*Δ and *sch9*Δ*vma2*Δ strains. Samples pre-grown on medium without acetic acid were diluted to OD_600nm_ 0.1 in medium buffered at pH 5 and containing 0, 2, 20, or 150 mM acetic acid on time point 0 (TP0). Then, the OD_600nm_ was measured after 6, 24 and 48 h and 6 and 12 days. Results are shown as mean ± SD of three biological replicates (*n* = 3).

Next, we analyzed the repercussions of acetic acid addition on growth and cell metabolism in the different yeast strains. This analysis was done in medium buffered at pH 5 to rule out strain-dependent differences in medium acidification and acid stress [32]. Although a difference was noticed for the growth rate and the cell density at which the cultures of the mutant strains entered the post-diauxic phase, addition of physiological amounts of acetic acid (2 and 20 mM) did not induce major growth defects. In contrast, addition of 150 mM acetic acid to the medium clearly decreased growth rate and maximum culture density in all tested yeast strains, and it also extended the lag phase of the *vma2*Δ and *sch9*Δ*vma2*Δ strain significantly (Figure 4). Hence, our data indicate that the V-ATPase fulfils an important role in dealing with the adaptation to extreme acid stress, which is in line with previous reports [17,18].

**Figure 5:**
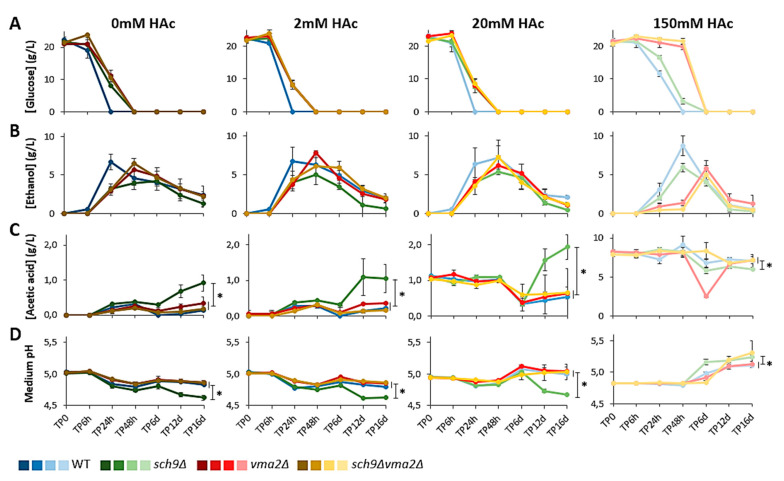
Accumulation of metabolites in the medium and medium pH over time. Samples pre-grown on medium without acetic acid were diluted to OD_600nm_ 0.1 in medium buffered at pH 5 and containing 0, 2, 20, or 150 mM acetic acid (HAc) on time point 0 (TP0). After 6, 24 and 48 h or 6, 12 and 16 days, samples were taken and the concentrations of glucose (**A**), ethanol (**B**) and acetic acid (**C**) present in the medium were measured by HPLC. The pH of the medium was then measured (**D**), revealing that the accumulation of acetic acid in the medium coincides with medium acidification, while acetic acid uptake from the medium coincides with medium alkalization. Results are shown as mean ± SD of 3 biological replicates (*n* = 3), * *p* < 0.05 as tested with ANOVA. The data are given for the BY4741 WT (blue) and the mutants *sch9*Δ (green), *vma2*Δ (red), *sch9*Δ*vma2*Δ (ochre).

We then monitored the consumption of glucose and the concentrations of ethanol and acetic acid in the medium over time. Based on the data from the control condition (0 mM acetic acid), different metabolic phases can be discriminated (Figure 5A–C). The first phase is characterized by the consumption of glucose and the accumulation of ethanol, as expected during fermentative growth of *S. cerevisiae*. Consistent with the slower growth of the mutants, a delay in glucose consumption and ethanol accumulation was observed for the mutant strains as compared to the WT strain. During this first phase the cultures also accumulated a small amount of acetic acid in the medium but, interestingly, here no apparent delay was seen and the acetic acid peaked simultaneously in all of the strains. This suggests that all strains first invest to replenish their pools of acetyl-CoA, the activated form of acetic acid, as this is required to support their exponential growth. The second phase is marked by the gradual uptake of ethanol and acetic acid from the medium. While ethanol was further consumed until the end of the experiment, acetic acid started to accumulate again, which we indicated as the start of the third metabolic phase. In general, the growth and metabolic patterns for each strain grown in medium containing 2 mM or 20 mM acetic acid were still comparable to the 0 mM control condition, while a significant deviation is seen when the strains were cultured in the presence of 150 mM acetic acid, especially in case of the *vma2*Δ and *sch9*Δ*vma2*Δ mutants.

### 3.4. The sch9Δ Strain Secretes More Acetic Acid

As mentioned, our analysis revealed a slight accumulation of acetic acid during the first phase of fermentative growth when cells were supplemented with medium containing 0 mM and 2 mM acetic acid. Overall, in these conditions all strains displayed similar levels of acetic acid accumulation of approximately 0.3 g/L (5 mM) (Figure 5C). This is in line with a previous study that reported 5 mM acetic acid accumulation for cells grown in medium supplemented with 2% glucose and buffered at pH 6 [11]. As the accumulation of acetic acid during fermentative growth did not exceed 0.5 g/L (8 mM) in the 0 mM and 2 mM acetic acid conditions, it is not surprising that no additional acetic acid accumulation in the medium was noticed when the start concentration of acetic acid already surpasses 0.5 g/L (8 mM), i.e., when cells were cultured in medium supplemented with 20 mM or 150 mM acetic acid.

After the initial accumulation of acetic acid in the medium, all strains in all conditions started to consume this acetic acid during the second phase, which in our assays mostly took place between 48 h and 6 days (Figure 5C). This is linked to the exhaustion of glucose (Figure 5A) and the de-repression of proteins required for acetate import and metabolization when the cells had traversed the diauxic shift [2; 3]. Nevertheless, the uptake of acetic acid did not coincide with an increase in culture density (Figure 4), indicative that the metabolization of acetic acid does not lead to a significant production of biomass. The extent of cellular acetate consumption seemed, however, to be dependent on the strain and the condition. The WT, *vma2*Δ and *sch9*Δ*vma2*Δ strains completely depleted the previously accumulated acetic acid in the 0 mM control condition and upon supplementation of 2 mM acetic acid. In the *sch9*Δ cells, the consumption of acetic acid was more limited under these conditions. When grown in medium containing 20 mM acetic acid, all strains, including the *sch9*Δ strain, consumed comparable amounts of acetic acid (±15 mM) (Figure 5C).

In the third phase, when cells were about six days in culture, the acetic acid levels in the medium started to accumulate again. Strikingly, this accumulation was approximately twice as high in the culture of the *sch9*Δ strain as compared to the cultures of the WT, the *vma2*Δ and the *sch9*Δ*vma2*Δ strain, and it reached a concentration of approximately 1 g/L after 16 days in both the 0 mM and 2 mM acetic acid conditions and almost 2 g/L in the 20 mM condition (Figure 5C). Interestingly, our data clearly indicated that the *sch9*Δ mutant requires a functional V-ATPase in order to produce and secrete significant amounts of acetic acid. Moreover, when cultured in the presence of 150 mM acetic acid, the *sch9*Δ cells were also unable to secrete and accumulate acetic acid in the medium. In fact, in this condition the medium acetate concentrations were slightly, but still significantly, lower in the *sch9*Δ mutant after 16 days of growth as compared to the other strains, indicative that the *sch9*Δ mutant consumes rather than produces acetic acid. This suggests a difference in the metabolic state of the *sch9*Δ cells when their culture medium is supplemented with high concentrations of acetic acid, which induces an extremely high acid stress condition.

As expected, the concentrations of acetic acid in the medium were nicely mirrored by the pH of the medium (Figure 5C,D). Indeed, the phases of acetic acid accumulation coincided with extracellular acidification, while the acetic acid consumption phase was accompanied by the alkalinization of the medium. Consistent with the increased acetic acid concentrations in the medium, the *sch9*Δ mutant was again the outlier, since the extracellular pH was always more acidic over the course of the experiment when cultured in medium without or with low concentrations of (2 mM or 20 mM) acetic acid. In contrast, no such difference was seen for the *sch9*Δ mutant in medium containing 150 mM acetic acid. In this condition, the *sch9*Δ strain behaved similar to the other strains, showing a gradual alkalinization from approximately pH 4.8 (at the start of the culture) to a pH above 5 by day 12. Albeit this alkalinization correlated to a reduced acetic acid concentrations in the medium (Figure 5C,D), it should be noted that the absolute acetic acid concentrations were still high, indicative that the observed reduction is not sufficient to explain the change in pH and that the latter is also a specific consequence of the loss of Sch9. In fact, also the *sch9*Δ*vma2*Δ displayed a more pronounced alkalinization when cultured for 12 days and beyond, even though no marked reduction of the acetic acid concentration in the medium was observed with this mutant.

### 3.5. Cytoplasmic pH Correlates to Growth

**Figure 6:**
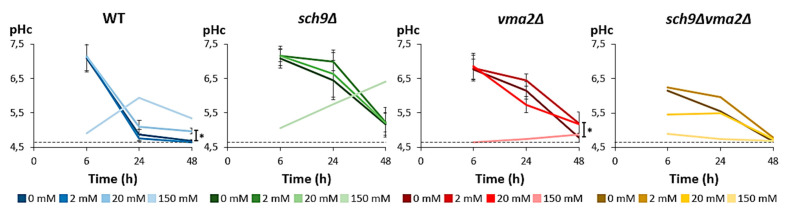
Cytosolic pH correlates with growth rate. Cultures of the BY4741 WT (blue) and the mutants *sch9*Δ (green), *vma2*Δ (red), *sch9*Δ*vma2*Δ (ochre) pre-grown on medium without acetic acid were diluted to OD_600_ 0.1 in medium buffered at pH 5 and containing 0, 2, 20, or 150 mM acetic acid on time point 0 (TP0). At the indicated time intervals, intracellular pH was measured using the ratiometric pH-sensor pHluorin. Results are shown as mean ± SD of 3 biological replicates (*n* = 3), * *p* < 0.05 as tested with ANOVA. For the *sch9*Δ*vma2*Δ strain, only 1 replicate is shown due to unreliable measurements of the other biological replicates. The dashed line indicates the lower detection limit for an accurate determination of cytosolic pH (pHc) with pHluorin as determined by internal calibration. Resulting pH values <4.65 were consequently corrected to pH 4.65.

Besides the extracellular pH, we also monitored the pH of the cytoplasm (pHc) in the different strains when grown on medium containing 0, 2, 20, or 150 mM acetic acid (Figure 6). It is known that during fermentative growth the pHc is mainly determined by the activity of Pma1, which is activated in the presence of glucose [56,57]. However, it is also established that the glucose-induced activation of Pma1 is compromised in cells carrying a deletion of *VMA2* [58]. Consistently, we noted that the cytoplasm of the *vma2*Δ and *sch9*Δ*vma2*Δ mutants was more acidic than that of the WT or the *sch9*Δ when measured after 6 h of growth. Interestingly, the pHc did not differ between the different growth conditions at this time point, except when the high concentration of 150 mM acetic acid was supplemented to the medium. The latter will be further discussed below. After 24 h of growth without or with low concentrations of acetic acid (2 mM or 20 mM), the WT strain was already in the post-diauxic phase displaying maximal OD_600nm_ levels (Figure 4) and this coincided with a drop of the pHc to approximately pH 5, which is consistent with previously reported data [41,59,60,61]. At this time point, the *sch9*Δ, *vma2*Δ and *sch9*Δ*vma2*Δ cells were still actively growing and their pHc values remained close to neutrality. Nonetheless, the cytoplasm also became acidic in these strains after 48 h of growth. Thus, pHc and growth generally correlated well in the conditions of 0, 2, and 20 mM acetic acid supplementation. Note that the pHc in the post-diauxic phase (48 h of growth) remained slightly more alkaline in the *sch9*Δ mutant under all conditions, while this was only the case in the WT strain when grown in the presence of 20 mM acetic acid and in the *vma2*Δ strain when grown in the presence of 2 mM or 20 mM acetic acid.

The pHc profile was severely disrupted in all of the strains when the growth medium contained 150 mM acetic acid. Especially in the *vma2*Δ and *sch9*Δ*vma2*Δ strains, which only started to resume growth after 48 h in this condition (Figure 4), the pHc remained low (<5). In the WT and *sch9*Δ strains, which displayed much shorter lag phases, a modest increase in pHc was observed, and this peaked after 24 h in the WT strain but steadily continued to increase in the *sch9*Δ mutant over the period of 48 h.

To further document the changes in pHc and determine whether the growth retardation seen in the presence of 150 mM acetic acid can be explained by a massive acetic acid influx via diffusion over the plasma membrane, we also monitored the cytosolic pH when acetic acid was given to cells during exponential growth (Supplemental Figure S3). As expected, the results 2 mM and 20 mM acetic acid correlated well with our data on acetic acid diffusion (Figure 2A,B), but most importantly, they confirmed that cells were able to maintain the pHc changes within limits. In contrast, however, the addition of 150 mM acetic acid indeed led to a severe acidification of the cytosol, which likely hampers several enzymes and other function required to support normal growth.

### 3.6. The Addition of 20 mM Acetic Acid Increases Chronological Lifespan

**Figure 7:**
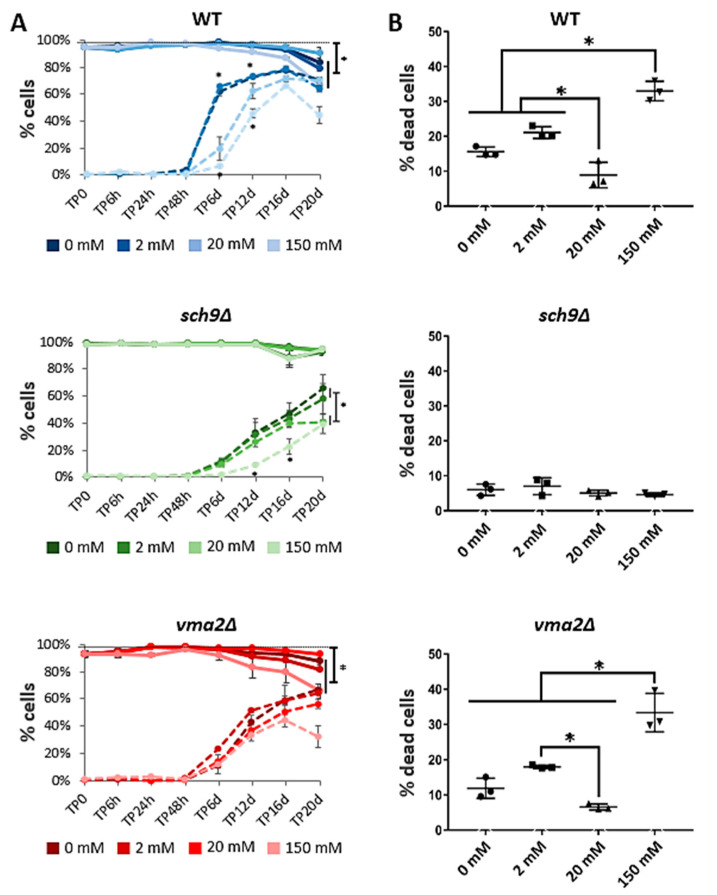
The effect of acetic acid on lifespan and ROS accumulation in WT, *sch9*Δ and *vma2*Δ strains. (**A**) Samples pre-grown on medium without acetic acid were diluted to OD_600_ 0.1 in medium buffered at pH 5 and containing 0, 2, 20, or 150 mM acetic acid on time point 0 (TP0). After 6, 24 and 48 h or 6, 12, 16 and 20 days, samples were taken to assess lifespan (full lines) and ROS accumulation (dashed lines) using SYTOX^TM^ green and DHE staining respectively. After 20 days, a significant amount of WT and *vma2*Δ had died in the 0, 2, and 150 mM acetic acid condition, when compared to the maximum number of viable cells throughout the experiment (indicated by the black dotted line), but not in the 20 mM condition. (**B**) Statistical differences between the acetic acid conditions were tested at the end-point of the measurements, after 20 days. Results are shown as mean ± SD of 3 biological replicates (*n* = 3), * *p* < 0.05 as tested with ANOVA.

Next, we analyzed the effects of acetic acid supplementation on cellular longevity. To this end, we monitored the chronological lifespan and the cellular accumulation of ROS by flow cytometry analysis, using SYTOX and DHE staining respectively (Figure 7). As this study aims to expose long-term effects of acetic acid treatment, the possibility that the tested acetic acid concentrations would induce immediate cell death must be ruled out. Therefore, the monitoring of cell viability was started immediately after dilution in the acetic acid-containing medium (0, 2, 20, or 150 mM). No immediate cell death was observed upon the addition of acetic acid to the medium for the WT, *sch9*Δ and *vma2*Δ strains, as shown in the overview figure (Supplemental Figure S4). In contrast, we noticed that approximately 40% of the *sch9*Δ*vma2*Δ cells were already dead at the start of the experiment, independent of the amount of acetic acid in the medium. It was indeed published that the *sch9*Δ*vma2*Δ strain has a synthetic sick phenotype characterized by a dramatically reduced survival in the post-diauxic and stationary phase [41], which explains the high ratio of cell death in this mutant. Moreover, while the WT, *sch9*Δ and *vma2*Δ strains displayed a gradual decrease in lifespan, we noticed that the *sch9*Δ*vma2*Δ cells manifested fluctuations. We believe these fluctuations are a result of emerging subpopulations containing secondary suppressor mutations and therefore this strain was excluded from further analysis.

The WT, *sch9*Δ and *vma2*Δ strains only showed very limited cell death during the 20 days that lifespan was monitored (Figure 7), probably due to the excellent buffering capacity of the phthalate buffer that was used to adjust the medium to pH 5. Still, by the end of the experiment (20 days), a significant number of cells died in the 0 mM and 2 mM acetic acid condition (Supplemental Figure S4). The detailed over-time analysis of the WT strain revealed a rapid increase of ROS and DHE-positive cells shortly after reaching the post-diauxic phase in the 0 mM and 2 mM acetic acid conditions. In the 20 mM and 150 mM conditions, on the other hand, a more gradual increase of ROS accumulating cells was noticed (Figure 7A), which might suggest that under these conditions the cells enhanced the activation of ROS coping strategies. The rate and extent of ROS accumulation in the WT strain did not strictly correlate with chronological lifespan since at the end of the experiment the amount of ROS was markedly lower in the 150 mM acetic acid condition, but still an increased number of WT cells died as compared to the 0, 2 mM and 20 mM acetic acid condition (Figure 7; Supplemental Figure S4). This is in line with previous reports, stating that high concentrations of acetic acid cause chronological aging [38]. Remarkably, however, we observed a significant reduction in cell death during growth in the presence of 20 mM acetic acid although this is also a higher-than-physiological concentration.

The data obtained for the *vma2*Δ strain is most similar to those of the WT strain, i.e., an extended chronological lifespan in the 20 mM acetic acid condition and a shortened chronological lifespan in the 150 mM acetic acid condition (Figure 7; Supplemental Figure S4). Yet, the increase in ROS over time was slower in the *vma2*Δ strain than in the WT strain in all acetic acid conditions, again showing that chronological lifespan and ROS accumulation do not strictly correlate.

In the long-lived *sch9*Δ strain, no significant cell death is noticed after 20 days, not even in the 150 mM acetic acid condition (Figure 7; Supplemental Figure S4). This is in line with previously published results, showing that stationary phase *sch9*Δ cells are more resistant to high concentrations of acetic acid [11]. As the transcription factors Gis1 and Msn2/4, which control the the expression of superoxide dismutases and catalases, are well-studied downstream targets that are under negative control of Sch9 [62,63], it is not surprising that the *sch9*Δ strain displays a general lower accumulation of DHE positive cells and an enhanced longevity. Still, when *sch9*Δ cells were grown in the presence of 150 mM acetic acid, a delay in ROS accumulation can be noticed, similar as observed in WT cells. Hence, this delay in ROS accumulation by high concentrations of acetic acid is at least in part independent of the Sch9-mediated changes in transcription via Gis1 and Msn2/4.

## 4. Discussion

### 4.1. A Functional V-ATPase Is Required to Counteract Short-Term Effects of Acetic Acid Stress

It is known that exposure of cells to sublethal concentrations of acetic acid evokes an extended lag phase that is followed by exponential growth at a lower specific growth rate [20]. It was then proposed that the prolonged lag phase is related to the time needed to adapt to the weak acid stress [20]. The passive diffusion of high amounts of acetic acid leads to inhibition of fermentative enzymes and an enhanced, but energy consuming, proton extrusion that is necessary to counteract intracellular acidification. Both processes are at the expense of biomass production [5,6,7,8,9,64]. Our data obtained upon addition of 150 mM acetic acid fit these previously reported observations as they confirmed an immediate intracellular acidification that is linked to a prolonged lag phase or slow growth phenotype in all strains tested. Furthermore, our data indicate that the V-ATPase plays a crucial role in cellular adaption to conditions of high acetic acid stress (150 mM). While all of the strains initially acidify comparably, the WT and *sch9*Δ strain recover much faster than the *vma2*Δ or *sch9*Δ*vma2*Δ strains. The cytosolic pH of strains lacking functional V-ATPase remains very acidic (<pH 5) during the tested time period (48 h) and correlates to the inability of these cells to resume growth in this timeframe. Our findings highlight the importance of the vacuolar and Golgi/endosomal V-ATPases in counteracting the intracellular pH changes induced by high concentrations of acetic acid [14,15,16]. Indeed, *VMA* mutants were also found to be more susceptible for intracellular pH changes and growth inhibition by acetic acid [12,14,17]. Even though this might be a direct effect of loss of V-ATPase function, it is also established that the glucose-induced activation of Pma1 is compromised in cells carrying a deletion of *VMA2* [58]. In this way, loss of functional V-ATPase might lead to the inability to fully activate Pma1, which is known to be the most important determinant of pHc during fermentative growth [56,57]. Notably, the V-ATPase has previously been shown to be important to allow growth in a variety of stress conditions [41,63,65,66]. This function of the V-ATPase has even been linked to its role in sphingolipid homeostasis [45].

Alternatively, the prolonged lag phase in the *vma2*Δ and *sch9*Δ*vma2*Δ strain could partially be explained by an increased diffusion of undissociated acetic acid over the membranes of these strains due to the observed changes in lipid-chain packing properties. As previously mentioned, membrane permeability for acetic acid is dependent on the physicochemical properties of the plasma membrane [55]. For instance, the more acetic acid-resistant yeast *Z. bailii* has a higher relative abundance of membrane sphingolipids, as well as longer sphingolipid and GPL chain lengths and less unsaturated fatty acids than *S. cerevisiae* [30]. Accordingly, upregulation of the unsaturation index of fatty acids in the plasma membrane has been linked to acetic acid tolerance in *S. cerevisiae* [29]. The stark reduction in fatty acid chain length and unsaturation index in the *vma2*Δ and *vma2*Δ*sch9*Δ strains may consequently partially underlie the higher susceptibility of these strains to high acetic acid concentrations. This hypothesis, however, remains questionable, as the higher initial passive diffusion of acetic acid over the membrane does not lead to overall higher cellular levels of acetic acid. On the contrary, reduced cytosolic steady state levels of acetic acid were observed in the *vma2*Δ and *vma2*Δ*sch9*Δ strains as well as the single *sch9*∆ mutant. Thus, this aspect remains to be clarified.

Despite the higher susceptibility to intracellular pH changes and the extension of the lag phase of the V-ATPase deficient strains grown on 150 mM of acetic acid in comparison to the WT strain, the *vma2*Δ strain behaved very similar to the WT strain when investigating chronological lifespan. In previous large-scale chronological survival studies, the *VMA* mutants have been identified as being short-lived [67,68]. This phenotype has been confirmed when *vma2*Δ cells are grown in unbuffered medium, but when grown on buffered medium, as is the case in our current experiment, the lifespan of *vma2*Δ cells increases significantly [41]. As such, the similar chronological lifespan of the *vma2*Δ and WT strain are in line with the expectations. Taken together, our results imply that even though a functional V-ATPase is required for early coping mechanisms to higher concentrations of acetic acid, it is not required for long-term acetic acid effects.

When lower concentrations of acetic acid (2 and 20 mM) were administered, the growth and metabolic patterns were comparable to those obtained for the control condition (0 mM acetic acid) in all strains, even the mutants lacking a functional V-ATPase, and this was paralleled by the ability of the cells to maintain the cytosolic pH around neutrality. As such, our results further corroborate the previously described relationship between cytosolic pH and specific growth rate [61].

### 4.2. The Long-Lived sch9Δ Strain Accumulates Acetic Acid and Triacylglycerol

We noticed a decrease chronological lifespan when cells were grown in medium containing 150 mM acetic acid, but an increased chronological survival when grown in medium containing 20 mM acetic acid. In both cases, a higher-than-physiological concentration was administered to the cells and thus at first sight the data may seem to be contradicting. However, also previous studies reported different outcomes for longevity. Indeed, one study reported a shortened chronological lifespan when cells were maintained in medium containing 10 mM acetic acid at pH 2.8 [11], while another study showed that addition of 10 mM acetic acid to medium at pH 3.7 does not affect chronological lifespan at all [38]. We believe that the different outcomes can be explained by differences the in experimental setup. One important factor is the pH. The previously reported experiments were performed at more acidic pH and although this eventually results in comparable concentrations of undissociated acid as in our experimental setup when using 20 mM acetic acid (±8 mM CH_3_COOH), the pH difference between extra- and intracellular pH is bigger in the previously published experiments, resulting in a higher driving force for inwards diffusion of acetic acid. In addition, in the study that observed enhanced cell death, the acetic acid concentration in the medium was kept constant by continuously resupplying the medium with fresh acetic acid [11]. As such, much more acetic acid was added to the medium by the end of the experiment (about 108.6 mM), which therefore is more comparable to our 150 mM setup where we also observed enhanced cell death.

Intriguingly, the long-lived *sch9*Δ strain produces and secretes itself high levels of acetic acid during the post-diauxic and stationary phase ranging from 1 g/L (20 mM) in the cultures without or with supplementation of 2 mM acetic acid to 2 g/L upon supplementation of 20 mM acetic acid. Since no such acetic acid accumulation is seen in the other strains, our data indicate that the *sch9*Δ strain exhibits a different metabolic mode, which is in line with the conclusion drawn from a previous study that analyzed the utilization of acetic acid in the *sch9*Δ strain [38]. Since we observed that addition of 20 mM acetic acid to the growth medium of WT and *vma2*Δ cells triggers a cytoprotective effect leading to their increased chronological survival, one may argue that the production and secretion of acetic acid by the *sch9*Δ cells is one of the reasons why the survival of these cells is similarly high under all conditions tested and thus independent of acetic acid supplementation. Indeed, it is plausible that an acetic acid concentration in the range of 20 mM, be it administered or secreted, is sufficient to induce an hormesis effect that allows the cells to cope better with the stresses associated to nutrient limitation and starvation. Careful comparison of our data (Figure 5 and Figure 7, and supplemental Figure S4) indicated that particularly during the period between 48 h and 6 days of culture cells consumed the extracellular acetic acid and this was accompanied by the appearance of cells with enhanced ROS levels. Upon deletion of *SCH9*, however, not all acetic acid is consumed and the ROS levels remain lower. A similar correlation is seen for WT cells and the *vma2*Δ cells when given at least 20 mM acetic acid. Then again not all of the acetic acid is consumed and ROS levels are lower. Obviously, upon administration of too much acetic acid, such as 150 mM, cells suffer from an overwhelming acid stress. Therefore, it would be interesting to study this in more detail as to determine the threshold concentration of acetic acid that would allow for an optimal hormesis effect.

Still, an intriguing question remains: what drives the production of acetic acid in post-diauxic and stationary *sch9*Δ cells? One possibility is that this relies on their enhanced reserves, including their increased TAG levels. Indeed, a recent study that examined the effect of weak acids in the haploid prototrophic *S. cerevisiae* strain CEN.PK 113-7D demonstrated that yeast cells exposed to acetic acid upregulate genes for the conversion of phosphatidic acid (PA) to TAG and downregulate genes for the synthesis of several GPLs [29], suggesting that cells are using less PA for GPL synthesis in favor of TAG synthesis. The role of TAG is pleiotropic and related to its function as a supplementary energy source and its role in lipid homeostasis that ensures the reproductive potential of cells [69]. Notably, yeast cells were shown to deplete their TAG reserves during growth on glucose in the presence of acetic acid, but to replenish these reserves after the diauxic shift [29]. Besides higher TAG levels, cells lacking Sch9 are also characterized by increased levels of the reserve carbohydrate and stress protectant trehalose. In fact, *sch9*Δ cells were shown to utilize acetic acid during aging for the storage of trehalose via a mechanism dependent on mitochondrial electron transport and acetate CoA transferase [38]. Hence, it would be interesting to monitor the levels of TAG and trehalose when *sch9*Δ cells are kept in nutrient-starved conditions for a more prolonged time to see whether these reserves are then being utilized to fuel again the production of acetic acid.

A comparison with the *sch9*Δ*vma2*Δ strain revealed that the *sch9*Δ strain is completely dependent on a functional V-ATPase for its acetic acid metabolism as well as its extended lifespan. From our observations, it cannot be deduced what exactly is the driving force for acetate secretion into the medium, and it would be interesting to elucidate how this relates to the V-ATPase functionality. Here, it should be noted that the second acetic acid accumulation phase occurs in post-diauxic cells where all of the glucose is consumed and where the proton pump Pma1 and the vacuolar V-ATPase are believed to be mostly inactive. Thus, it is unlikely that a proton gradient over the plasma membrane would be the main factor. However, it is known that the Golgi and endosomal V-ATPase is not regulated by glucose though its activity is still dependent on the membrane lipid composition [70,71,72]. Therefore, given the specific requirement of the V-ATPase for this accumulation of acetic acid in the medium, it is tempting to speculate that this relates to the Golgi and endosomal V-ATPase variant. In our experimental setup, the lifespan extension observed upon the addition of 20 mM acetic acid or by deletion of *SCH9* seems to be linked to cellular pH homeostasis in another way as well: both interventions result in a slightly more alkaline pHc in the post-diauxic phase. This seems to support the recent finding that a more alkaline starvation pHc is linked to increased chronological lifespan [73]. Still, the addition of 20 mM acetic acid to the *vma2*Δ strain also results in slight alkalinization and an increased survival, confirming the that the disruption of the vacuolar, Golgi and endosomal V-ATPases is not sufficient to significantly impact on the acetic acid effects when cells are being aged in medium buffered at pH 5, which is in line with previously reported observations [41]. Finally, we should mention that the V-ATPase is essential to maintain an optimal lipid homeostasis. Besides our observation that loss of a functional V-ATPases is associated with shorter fatty acid chain lengths and higher saturation of GPL and lyso-lipids, thereby affecting membrane thickness and permeability, we found *vma2*Δ and *sch9*Δ*vma2*Δ to display increased levels of ceramides. Hence, loss of the V-ATPase also affects the so-called sphingolipid rheostat, which is crucial for the regulation of stress tolerance and longevity of yeast cells, including *sch9*Δ cells [43].

In conclusion, our study advances our understanding of acetic acid stress and further details the physiological responses to acid stress. We demonstrated that a functional V-ATPase is required to allow for an optimal adaptation to acetic acid stress and to counteract its short-term effects. We provide evidence that the supplementation of 20 mM acetic acid has a cytoprotective and presumable hormesis effect that extends the longevity of all strains investigated. We also demonstrate that the long-lived *sch9*∆ strain produces and secretes itself significant amounts of acetic acid during stationary phase, which in addition to its enhanced accumulation of storage lipids underlies the increased chronological lifespan of this strain.

## Data Availability

All data are contained within the article and supplementary material. The raw data are available on request from the corresponding author.

## Supplementary Materials

The following are available online at https://www.mdpi.com/article/10.3390/microorganisms9091871/s1, Figure S1: Passive diffusion of C^14^-labelled acetic acid into yeast cells; Figure S2: Average acetic acid uptake rate over time normalized to dry weight; Figure S3: Addition of 20 and 150 mM of acetic acid induces a rapid acidification of the cytosol; Figure S4: Overview of cell death and ROS accumulation in WT, *sch9*Δ, *vma2*Δ*,* and *sch9*Δ*vma2*Δ cells during aging in the presence of acetic acid.
