# Supplementary material for: The Role of Sch9 and the V-ATPase in the Adaptation Response to Acetic Acid and the Consequences for Growth and Chronological Lifespan
Source: Microorganisms. 2021 Sep 3;9(9):1871. doi: 10.3390/microorganisms9091871 (PMC8472237; doi:10.3390/microorganisms9091871)

microorganisms

MDPI

Supplementary figures

The role of Sch9 and the V-ATPase in the adaptation response to acetic acid and the consequences for growth and chronological lifespan

Marie-Anne Deprez, Jeroen Maertens, Lisbeth Olssen, Maurizio Bettiga, Joris Winderickx

Figure S1: Passive diffusion of C14-labelled acetic acid into yeast cells. Cells expressing cytosolic pHluorin were grown to mid-log phase in medium without acetic acid. After a short starvation in buffer, 2 mM or 20 mM C^{14}-labelled acetic acid was pulsed to the cells (0 min) and the uptake was monitored during 10 min. Results are shown with 90% confidence intervals in dotted lines. The data are given for the BY4741 WT strain (blue lines) and the isogenic mutant strains *sch9Δ* (green lines), *vma2Δ* (red lines) and *sch9Δvma2Δ* (pink lines) as indicated.

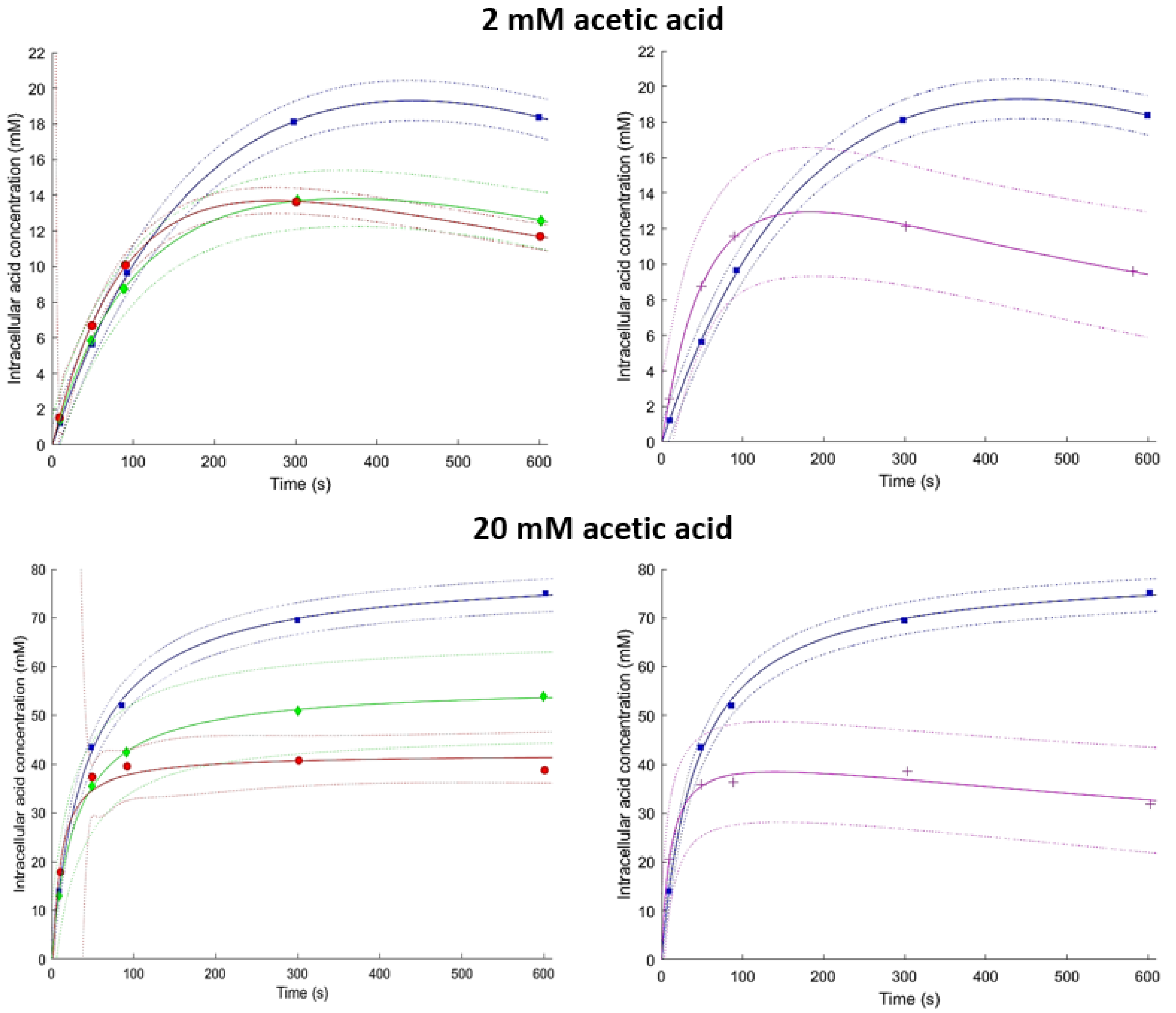

Figure S2: Average acetic acid uptake rate over time normalized to dry weight (dw). Results are shown with 95% confidence intervals in dotted lines. The data are given for the BY4741 WT strain (blue lines) and the isogenic mutant strains *sch9Δ* (green lines), *vma2Δ* (red lines) and *sch9Δvma2Δ* (ochre lines). The initial acetic acid uptake rate, as calculated during the first 10 seconds, was sightly higher in the *sch9Δ* strain when compared to the WT strain but this difference was not significantl. In contrast, the enhaced initial uptake rates observed in the *vma2Δ* and the *sch9Δvma2Δ* strains were significantly different from the WT stran with 90% and 95% confidence, respectively. σ; standard error of the mean.

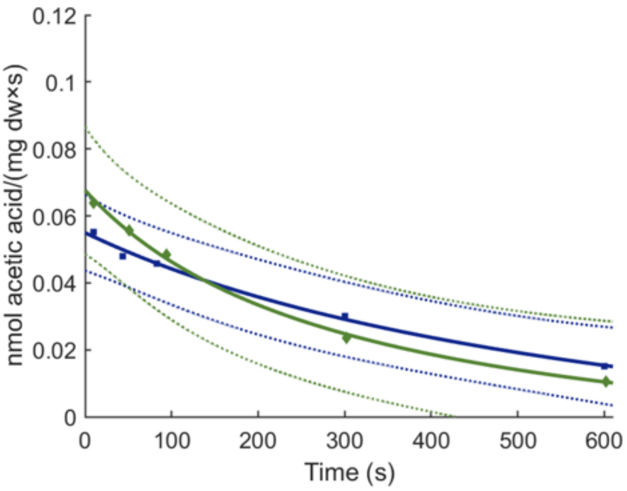

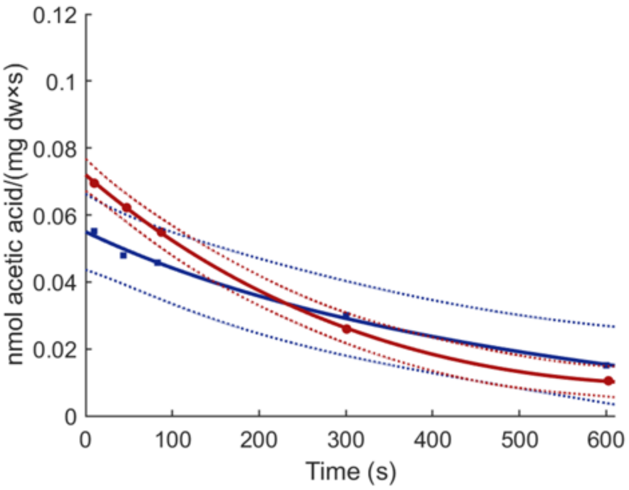

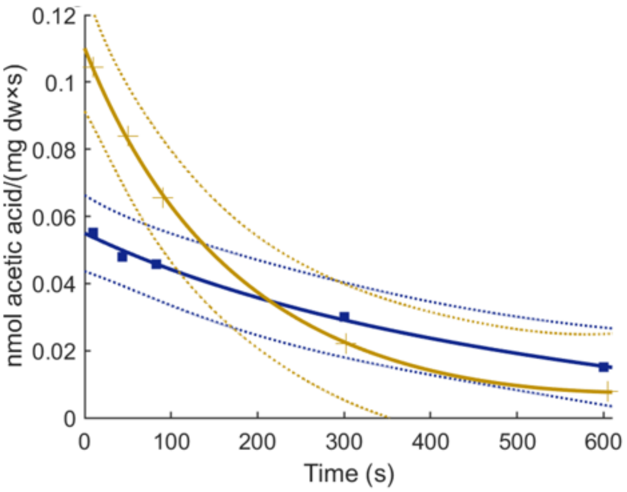

Initial uptake rate (uptake/s in first 10 sec, normalized to dry weight)		
	nmol acetic acid/(mg dw×s)	σ
**WT**	0.058	0.008
sch9Δ	0.064	0.017
vma2Δ	0.070	0.011
sch9Δvma2Δ	0.110	0.002

Figure S3: Addition of 20 and 150 mM of acetic acid induces a rapid acidification of the cytosol. Cells expressing cytosolic pHluorin were grown to mid-log phase in medium without acetic acid. After establishing the baseline cytosolic pH on growth medium, an equal volume of concentrated acetic acid was added to each culture to reach a final concentration of 0, 2, 20 or 150 mM acetic acid. The acetic acid pulse is indicated by the black arrow. Results are shown as mean ± SD of 3 biological replicates (N = 3). The data are given for the BY4741 WT (blue), the *sch9Δ* (green), the *vma2Δ* (red), and the *sch9Δvma2Δ* (ochre) strains as indicated.

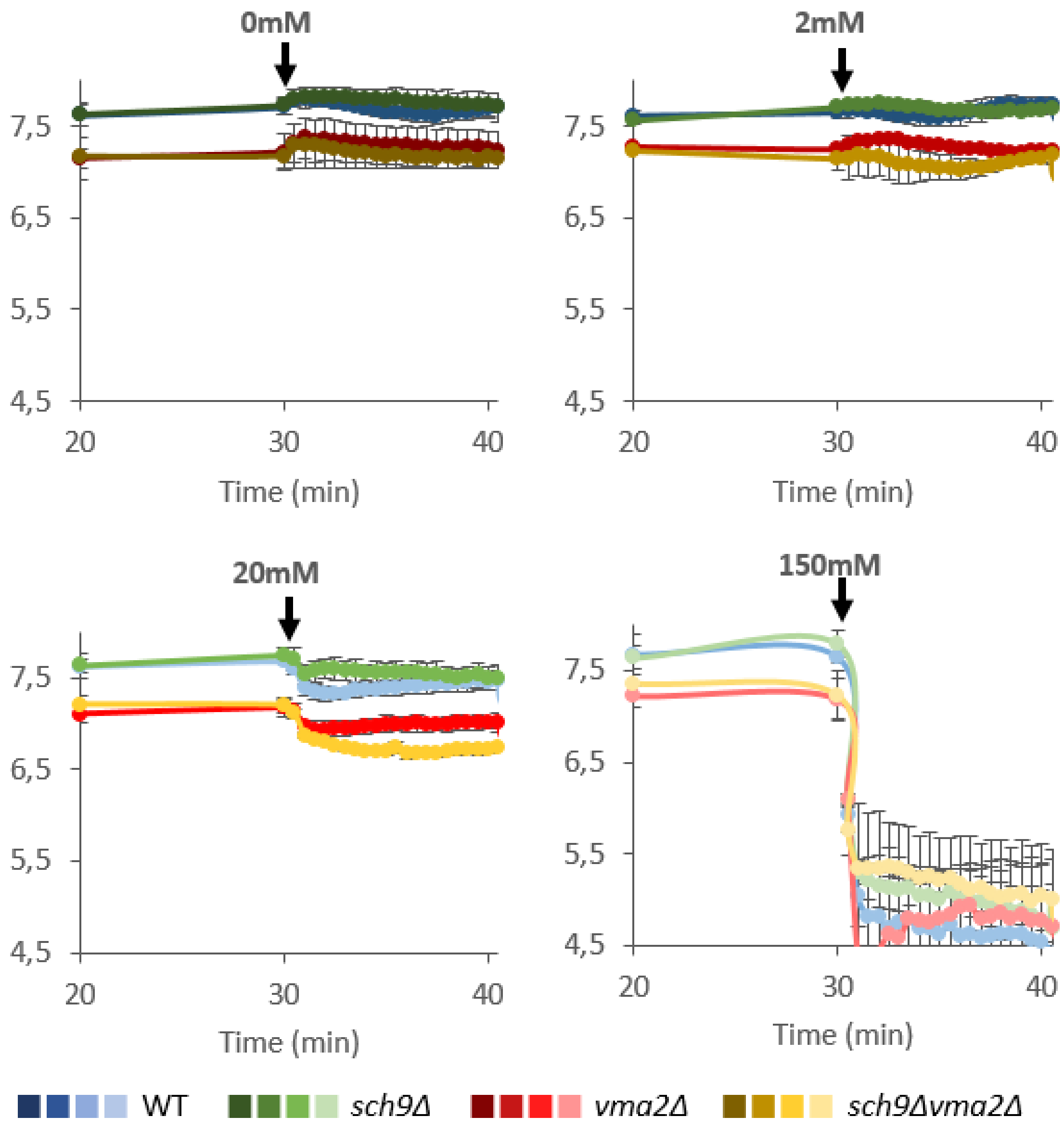

Figure S4: Overview of cell death and ROS accumulation in BY4741 WT, *sch9Δ*, *vma2Δ* and *sch9Δvma2Δ* cells during aging in the presence of acetic acid. Samples pre-grown on medium without acetic acid were diluted to OD_{600} 0.1 in medium (pH 5) containing 0, 2, 20 or 150 mM acetic acid (HAc) on time point 0 (TP0). After 6, 24 and 48 hours or 6, 12, 16 and 20 days, samples were taken to assess lifespan and ROS accumulation using SYTOX™ green and DHE staining respectively.

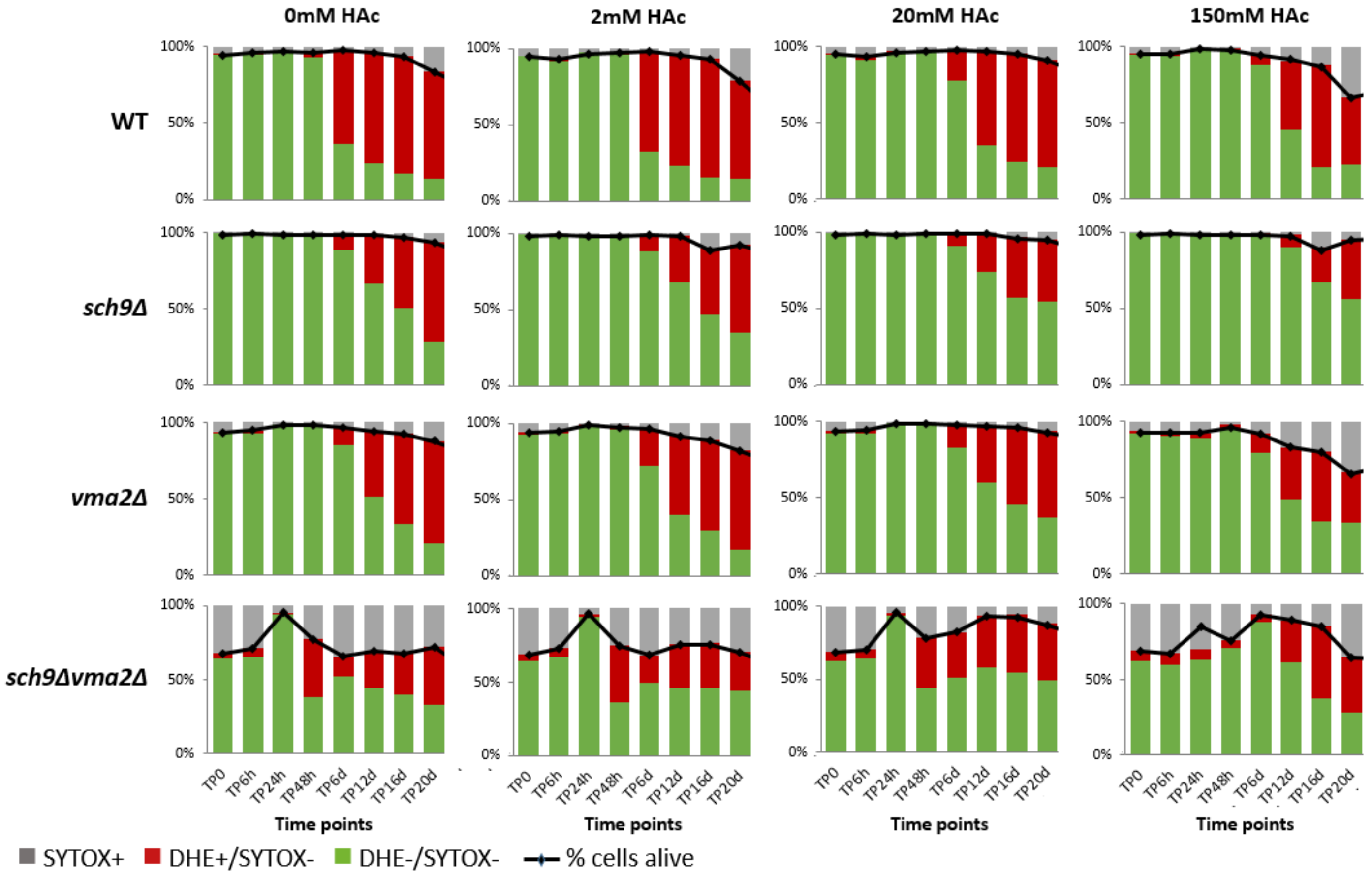

Supplement: Supplementary file 1 [file microorganisms-09-01871-s001.zip › microorganisms-1332042-supplementary.pdf]
